# Fully automated, level set-based segmentation for knee MRIs using an adaptive force function and template: data from the osteoarthritis initiative

**DOI:** 10.1186/s12938-016-0225-7

**Published:** 2016-08-24

**Authors:** Chunsoo Ahn, Toan Duc Bui, Yong-woo Lee, Jitae Shin, Hyunjin Park

**Affiliations:** School of Electronic and Electrical Engineering, Sungkyunkwan University, Suwon, South Korea

**Keywords:** Knee segmentation, Cartilage, Magnetic resonance imaging, Medical image processing

## Abstract

**Background:**

This study focuses on osteoarthritis (OA), which affects millions of adults and occurs in knee cartilage. Diagnosis of OA requires accurate segmentation of cartilage structures. Existing approaches to cartilage segmentation of knee imaging suffer from either lack of fully automatic algorithm, sub-par segmentation accuracy, or failure to consider all three cartilage tissues.

**Methods:**

We propose a novel segmentation algorithm for knee cartilages with level set-based segmentation method and novel template data. We used 20 normal subjects from osteoarthritis initiative database to construct new template data. We adopt spatial fuzzy C-mean clustering for automatic initialization of contours. Force function of our algorithm is modified to improve segmentation performance.

**Results:**

The proposed algorithm resulted in dice similarity coefficients (DSCs) of 87.1, 84.8 and 81.7 % for the femoral, patellar, and tibial cartilage, respectively from 10 subjects. The DSC results showed improvements of 8.8, 4.3 and 3.5 % for the femoral, patellar, and tibial cartilage respectively compared to existing approaches. Our algorithm could be applied to all three cartilage structures unlike existing approaches that considered only two cartilage tissues.

**Conclusions:**

Our study proposes a novel fully automated segmentation algorithm adapted for three types of knee cartilage tissues. We leverage state-of-the-art level set approach with newly constructed knee template. The experimental results show that the proposed method improves the performance by an average of 5 % over existing methods.

## Background

Segmentation is an especially important part of diagnostic medical imaging because it can separate abnormal regions from normal regions [1]. This study is focused on osteoarthritis (OA), a prevalent but poorly understood disease that affects millions of adults and occurs in the bone-like cartilage of the femur or tibia [2]. To diagnose OA, doctors must extract the cartilage, femur, or tibia from a time series of MRIs to determine their shape and size. Recently, previous works attempted to segment for knee magnetic resonance imaging (MRI) from various techniques; atlas-based segmentation [3–7] and others [8–11]. But the cartilage segmentation is difficult, because cartilage intensity varies, it is thin, and fat and muscle tissues encircle the cartilage boundary. Fat and muscle tissue in a knee MRI create abstract noise, which leads to holes or over-segmentation.

There have been several knee segmentation methods that are relevant to our work. Folkesson et al. [8] proposed the cartilage segmentation method for MRIs with two step multiclass classification scheme. In [8], they implemented a fully automatic cartilage segmentation in low-field MR scanners. Also, Grau et al. [3] used a novel modification of watershed transform, which led to better incorporation of prior information. Li et al. [9] proposed a non-model-based method based on novel multi-surface graph search algorithm for cartilage segmentation as a semi-automatic cartilage segmentation procedure. However, the above methods [3, 8, 9] doesnt include final separation, which means only femoral and tibial cartilage segmentation without patella was performed. Also, these methods reported poor performance of cartilage segmentation [3, 8, 9].

We summarized fully automatic segmentation methods with finial separation of knee cartilages as shown in Table 1. These studies have assessed fully automatic segmentation, and most methods studied cartilage segmentation with template images, since cartilage is the important tissue. The methods evaluated MRIs of various Tesla values; a higher Tesla value indicates less noise. We also focus on fully automated bone and cartilage segmentation with template images from knee MRIs with noise. Tamez-Pena et al. [6] proposed a fully automated knee segmentation procedure that could segment the femoral and tibial cartilage in a knee MRI scan without human intervention. They showed the relative accuracy of the volumetric measurements for the entire femur, the femoral trochlea, the central lateral femur, the posterior lateral femur, the medial tibia, and the lateral tibia. But fuzzy voting algorithm is one kind of heuristic methods, which is difficult to express by numerical verification. Shan et al. [5] proposed an automatic, atlas-based, three-label cartilage segmentation approach with a probabilistic classifier that created atlas images. They segmented the femoral and tibial cartilage, which have lower mean dice similarity coefficients (DSC) of 78.2 and 82.6 %, respectively, than [6]. A hybrid segmentation method [4] was proposed and based on pre-segmentation with a statistical shape model and a fine segmentation with a fast marching algorithm from knee CTs. They segmented the femur, tibia, and patella stably and with good accuracy. However, this method was not suitable for cartilage segmentation, because segmentation can fail for image data with locally weak bone edges. Ababneh et al. [7] proposed a new, fully automated, content-based system for knee bone segmentation from MRI. They used a content-based image block classification mechanism in conjunction with graph-cut methodology. The results showed an automatic bone detection rate of 0.99 and an average segmentation accuracy of 0.95 DSC. However, this segmentation is only suitable for knee bone, because it uses a content-based image block classification mechanism, which is not suitable for cartilage. Dodin et al. [10] developed a new automatic segmentation algorithm to quantify human knee cartilage volume from knee 3-D MR images that contain the bone-cartilage interfaces of the femur and tibia. They had validated results with DSCs of 0.84, 0.85 and 0.84 for the global, femoral, and tibial cartilage, respectively. They also proposed a fully automated bone segmentation method for the human knee (femur and tibia) from MRI based on the ray casting technique. This technique relies on the decomposition of the MR images into multiple surface layers to localize the bone boundaries, and several partial segmentation objects are automatically merged to obtain the final segmentation of the bones [11]. However, they focused on bone segmentation and did not include cartilage segmentation.

**Table 1:** Comparison of existing knee segmentation methods

Authors	Key algorithm	Segmentation tissues	Modalities	Tesla	Subject numbers	Template image
Tamez-Pena et al. [6]	Fuzzy voting algorithm	Femoral cartilage, tibial cartilage	T1-weighted MRI (3-D DESS WE)	3T	12	Used
Shan et al. [5]	kNN classification	Femoral cartilage, tibial cartilage, femur, tibia	T1-weighted and partially T2-weighted MRI	–	18	Used
Ringenbach et al. [4]	Fast marching algorithm (region growing)	Femur, tibia, patella	CT	–	20	Used
Ababneh et al. [7]	Graph-cut algorithm	Femur, tibia	T2-weighted MRI	3T	200 (14 slices per each subject)	Used
Dodin et al. [11]	Ray casting technique	Femur, tibia	T2-weighted MRI (3-D-FISP)	1.5T	161	Unused
Dodin et al. [10]	Bayesian decision criterion	Femoral cartilage, tibial cartilage	T1-weighted MRI (3-D DESS WE)	3T	14	Unused

In this paper, we propose a novel template-based knee segmentation method, based on level set algorithm, which is more accurate for the segmentation of all three cartilages. We focused on the level set segmentation algorithm and on parametric deformable models, because the level set segmentation algorithm can accommodate the variability of biological structures over time and across individuals. Level set segmentation can be divided into two different classes: edge-based [12] and region-based [13] segmentation. The edge-based model, which uses image gradient information to find contours with a force function, has advantages for use with inhomogeneous objects. However, it is sensitive to noise and requires a long computational time. In contrast, the region-based model, which deforms to minimize a given energy function, is less sensitive to the initial contour location than is the edge-based model and requires less computational time. The region-based model, however, cannot resolve segments in an inhomogeneous object. To segment an object with inhomogeneous properties, Lankton et al. proposed using localizing active contours segmentation [13]. However, localizing active contours is sensitive to the initial contour. If the initial position is far away from the objects, the model may have a local minima problem. On the other hand, the model can get a more accurate segmentation if the initial contour is closer to the genuine boundary.

In this paper, we propose a novel, template-based knee segmentation method that is more accurate for the segmentation of all three cartilages. The proposed method is based on a level set algorithm and creates an initial contour by Spatial Fuzzy C-Means (SFCM) [14] for a fully automatic algorithm. We have already investigated the acquisition of the automatic initial contour based on localizing region-based active contours in [15] and describe this procedure in the Initial contour subsection of the Methods section. Finally, we created a template data set and modified the force function in a level set algorithm, which can segment knee cartilage from knee bone.

## Methods

**Fig. 1:**
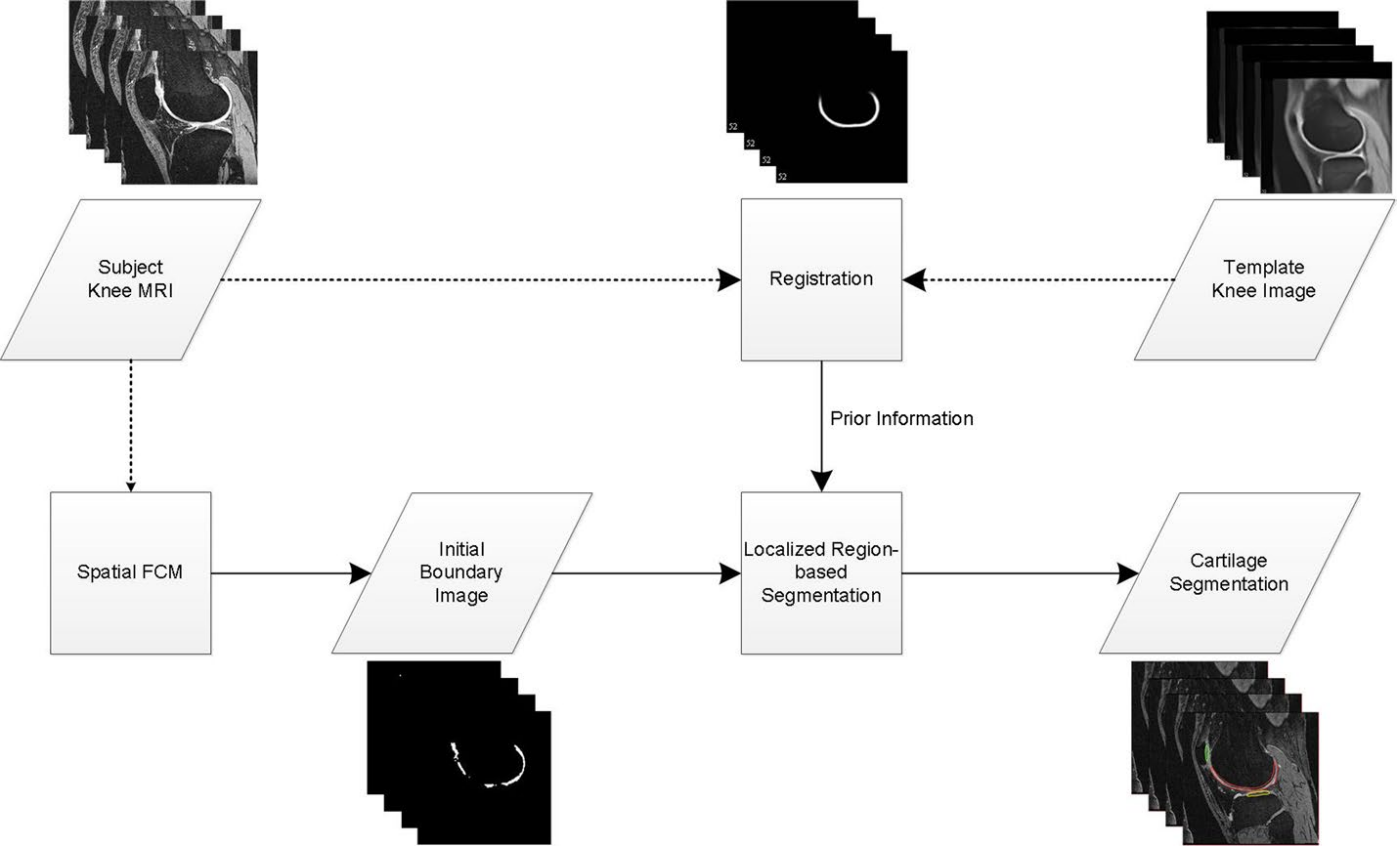
Proposed overall algorithm

Figure 1 shows the proposed overall algorithm. Our method operates in two phases: (1) decide the initial contour; and (2) apply the level-set method with modified localizing region-based active contours. The initial contour is moved toward outside or inside by local energy. The contour is moved by minimizing the local energy with the proposed energy model. We modified localizing region-based active contour model. The template improves the procedures in both phases. The approach is applied to three structures, namely femoral, tibial, and patella cartilage, respectively. The template provides initial contours for three cartilage structures separately. Thus, each cartilage segmentation starts with respective initial contour and the contour is optimized by our proposed local energy model.

We used knee MRI data from the OAI (The Osteoarthritis Initiative, http://www.oai.ucsf.edu) [16] and created template images to calculate the initial contour by SFCM. In calculating the initial contour, we approximated the initial boundary via modified SFCM. The prior information is generated image registration, which aligns each subjects knee MR image with the template data. Next, we applied the segmentation by localizing active contours with the newly modified force function. The fully automatic processes yielded segmented images of the femur, tibia, femoral cartilage, and tibial cartilage. In this section, we explain the proposed segmentation method in detail.

## MRI acquisition

The MRI exams consisted of testretest acquisitions on a 3-T MRI system (Siemens Magnetom Trio, Erlangen, Germany) with a quadrature transmitting-receiving knee coil (USA Instruments, Aurora, OH, USA) [4]. We used MR images from participants randomly chosen from the OAI database. We employed a 3-D dual echo steady state (DESS) sequence with water excitation (WE), and the images were acquired with a $384 \times 384$ matrix (0.365-mm in-plane resolution) and a slice thickness of 0.7 mm. These 3-D DESS WE image series have been used previously [16].

## Knee template

**Fig. 2:**
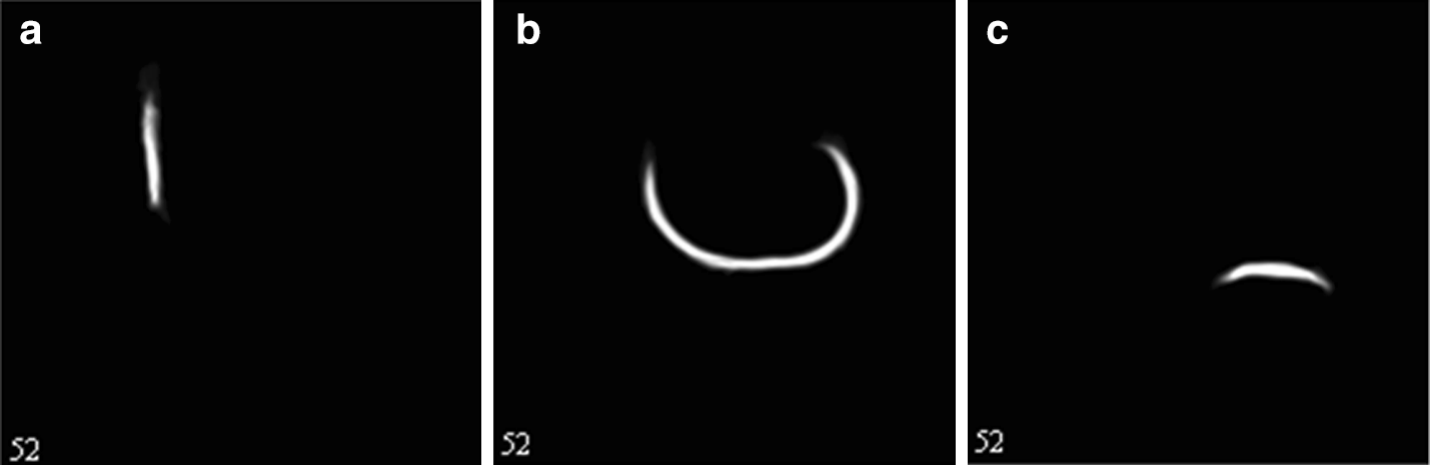
Knee template images. **a** Patellar cartilage, **b** femoral cartilage, **c** tibial cartilage

We used 20 normal subjects from the OAI database ranging in age from 40 to 79 years, divided into four age groups (4049, 5059, 6069 and ≥70). We applied group-wise registration, Symmetric Group-wise Normalization (SyGN) [17], to form the template data. The resulting template images are shown in Fig. 2. A probabilistic template provided the spatially dependent prior, P(l), for the segmentation method. This allowed us to restrict the segmentation to the ROI, helped to minimize the influence of noise, and improved segmentation robustness. The knee templates were obtained through three-label segmentations into categories such as femoral, tibial, and patellar cartilage. The templates were derived from 3-D DESS WE series from 20 subjects with visually normal knee images. Each template is made by 5120 slices because one subject has 256 slices. The series for these templates were selected from the public release OAI image dataset 0.E.1 and were from the baseline pilot study. We used template images to calculate the initial contour in the level set algorithm, and the template images were also applied to the new energy function.

## Initial contour

Traditional fuzzy C-mean clustering (FCM) is one of the most widely applied methods in medical image segmentation [12]. The aim of this method is to classify image into clusters by minimizing an objective function as follows:

1 $$\begin{aligned} J_{FCM}=\sum _{j=1}^{N}\sum _{i=1}^{c} u^{m}_{ij}||x_j-v_i ||^2 \end{aligned}$$

where $X=(x_1,x_2,...,x_N)$ is an image with *N* pixels, *c* is the number of clusters with $2 \le c \le n-1$ , and $v_i$ is the $i{th}$ cluster center. The parameter $m>1$ , which is set to 2 in this paper, is a parameter to control the fuzziness of the result. The membership function $u_{ij}$ , which indicates the degree of membership of the $j{th}$ object to the $i{th}$ cluster, is constrained as follows:

2 $$\begin{aligned} \sum _{i=1}^{c} u_{ij}=1,\quad0 \le u_{ij} \le 1 \end{aligned}$$

The membership function, $u_{ij},$ and centroids, $v_i,$ are updated iteratively:

3 $$\begin{aligned} u_{ij}=\frac{1}{{{\sum \nolimits _{k=1}^{c}}} \biggl ( \frac{||x_j-v_i ||^2}{||x_j-v_k ||^2}\biggr ) ^\frac{2}{m-1}} \end{aligned}$$

4 $$\begin{aligned} v_i=\frac{\sum _{j=1}^{n} u_{ji}^m x_j}{\sum _{j=1}^{n} u_{ji}^m} \end{aligned}$$

Although FCM is an unsupervised technique and is useful for clustering methods, it does not consider spatial information. Hence, it fails to segment images in the presence of noise. Chuang et al. [14] proposed a way to incorporate spatial information into the membership function for clustering as follows:

5 $$\begin{aligned} u_{ij}=\frac{ u_{ij}^p h_{ij}^q}{\sum _{k=1}^{c} u_{kj}^p h_{kj}^q} \end{aligned}$$

where *p* and *q* are parameters, which are set to 1 in this paper, to control the relative importance of both functions. Spatial information, $h_{ij}$, is defined as:

6 $$\begin{aligned} h_{ij}=\sum _{k \in NB(x_j)} u_{ik} \end{aligned}$$

where $NB(x_j)$ denotes a square window centered on pixel $x_j$ in the spatial domain. The membership function, $u_{ij}$, and centroids, $v_i$, are recalculated with Eqs. (3) and (4).

By using spatial information, the method improves the segmentation results. These results show the effect of noise in this segmentation method. We consider this method to be a pre-processing step to approximate cartilage contours.

## Modified localizing region-based active contours

The segmentation by localizing region-based active contours is a model-based technique that can be used with a suitable volumetric (or 3-D) image and that is insensitive to noise. The localizing region-based active contours algorithm is not based on global region models. Instead, it allows the foreground and background to be described in terms of smaller local regions, removing the assumption that the foreground and background regions can be represented with global statistics. Analyzing local regions leads to the construction of a family of local energies at each point along the curve. To optimize these local energies, each point is considered separately and moved to minimize (or maximize) the energy computed in its local region. To compute these local energies, local neighborhoods are split into local interiors and local exteriors by the evolving curve. The energy is then optimized by fitting a model to each local region. Our proposed model implements segmentation by minimizing the following energy function:

7 $$\begin{aligned} \begin{aligned} E\bigl (\phi \bigr )&= \lambda \int _{\Omega _x} \delta \bigl (\phi (x)\bigr ) ||\nabla \phi (x)||\,dx \\&\quad+ \int _{\Omega _x} \delta \bigl (\phi (x)\bigr ) \int _{\Omega _y} K_\sigma (x,y) F_{new}\bigl (I(y), \phi (y) \bigr ) \,dydx\\ \end{aligned} \end{aligned}$$

where $\Omega$ is an image domain with independent spatial variables *x* and *y*, and the smooth Dirac delta function, $\delta$, is defined as in [13]:

8 $$\begin{aligned} \delta \left( \phi (x)\right) = {\left\{ \begin{array}{ll} 1, &{} \quad\phi (x)=0 \\ 0, &{} \quad|\phi (x)| < \varepsilon \\ \frac{1}{2\varepsilon } \bigl \{ 1+ \cos \bigl ( \frac{\pi \phi (x) }{\varepsilon }\bigr )\bigr \}, &{} \quad\text {otherwise} \end{array}\right. } \end{aligned}$$

In addition, *I*(*x*) is the intensity of pixel on the domain $\Omega$, $\phi (x)$ is a signed distance function while $\phi (x)=0$ is a set of contour pixels, $K_\sigma$ is a truncated Gaussian kernel with scale $\sigma,$ which is used to remove some noise in local regions, and $F_{new}$ is an internal energy measure used to express local adherence to the given model at each point along the contour. $F_{new}$ is represented by:

9 $$\begin{aligned} F_{new}\bigl (I(y), \phi (y) \bigr )=F\bigl (I(y), \phi (y) \bigr )+\beta F_{template}+ \alpha F_{penalty} \end{aligned}$$

where the proposed $F_{template}$ and $F_{penalty}$ terms are explained more clearly in the subsection for Knee template and Eq. (14), respectively. *F* is an internal energy measure used to express the local adherence to a given model at each point along the contour and is represented by [13]:

10 $$\begin{aligned} F \bigl (I(y), \phi (y) \bigr ) = {\left\{ \begin{array}{ll} H \bigl ( \phi (y) \bigr ) \bigl (I(y) -u(x)\bigr )^2 \\+\bigl (\ 1- H \bigl ( \phi (y) \bigr ) \bigr ) \bigl (I(y) -v(x)\bigr )^2 , &\quad\text {for C-V feature}\\ \bigl ( v(x)-u(x)\bigr )^2&\quad \text {for Yezzi feature} \end{array}\right. } \end{aligned}$$

where *H* is the Heaviside function to specify the interior of the contour:

11 $$\begin{aligned} H \left( \phi (y)\right) = {\left\{ \begin{array}{ll} 1, &\quad \phi (y) < -\varepsilon \\ 0, &\quad \phi (y) > \varepsilon \\ \frac{1}{2} \left \{ 1+ \frac{1}{\varepsilon }+ \frac{1}{\pi }\sin \left ( \frac{\pi \phi (y) }{\varepsilon }\right )\right \}, &\quad \text {otherwise} \end{array}\right. } \end{aligned}$$

The local mean intensities of the interior function, *u*(*x*), and exterior function, *v*(*x*), are obtained as:

12 $$\begin{aligned} u(x) =\frac{\int _{\Omega } K_\sigma H \bigl (\phi (y) \bigr ) I(y) \,dy}{\int _{\Omega } K_\sigma H \bigl (\phi (y) \bigr )\,dy} \end{aligned}$$

13 $$\begin{aligned} v(x) =\frac{\int _{\Omega } K_\sigma \bigl ( 1- H \bigl (\phi (y) \bigr ) \bigr ) I(y) \,dy}{\int _{\Omega } K_\sigma \bigl ( 1- H \bigl (\phi (y) \bigr ) \bigr ) \,dy} \end{aligned}$$

$F_{template}$, the second term in Eq. (9), which has already been obtained from the prior information of knee template data in the subsection for Knee template, is an energy function of the knee template, and $F_{penalty}$, the third term in Eq. (9), is a novel term that is incorporated into the fitting term. $F_{penalty}$ is designed for pixels that do not belong to the regional subject. The penalty term increases to 1 as the pixel intensity moves away from the region, and equals zero within the region. Hence, the penalty term is defined as:

14 $$\begin{aligned} F_{penalty} = {\left\{ \begin{array}{ll} 0, &{} \quad x \in [T_{low}, T_{high}] \\ \max \biggl ( \frac{T_{low}-I(x)}{T_{low}}, \frac{I(x)-T_{high}}{I(x)}\biggr ), &{} \quad\text {otherwise} \end{array}\right. } \end{aligned}$$

where $T_{low}$ and $T_{high}$ are the low and high thresholds, respectively.

The probability density of subject intensity usually follows a Gaussian distribution with mean $\mu$ and standard deviation $\sigma$. Hence, $T_{low}$ and $T_{high}$ are defined as:

15 $$\begin{aligned} T_{low} = \mu -k\sigma ; \qquad T_{high} = \mu +k\sigma \end{aligned}$$

where $\mu =\frac{1}{n}\sum _{i=1}^{n}I(x_i)$, $\sigma ^2=\frac{1}{n-1}\sum _{i=1}^{n} ( I(x_i) -\mu)^2$, *n* is the number of pixels in the subject samples, and *k* is a factor determining the confidence level. $T_{low}$ and $T_{high}$ determine the valid subject region. We define the pixel sign as $-1$ for areas outside the region and 1 for areas inside the region:

16 $$\begin{aligned} sign(x)= {\left\{ \begin{array}{ll} 1, &{} \quad x \in [T_{low}, T_{high}] \\ -1, &{} \quad \text {otherwise} \end{array}\right. } \Leftrightarrow sign(x)=\bigl (I(x)-T_{low}\bigr ) \bigl (T_{high}-I(x)\bigr ) \end{aligned}$$

From Eqs. (14) and (16), $F_{penalty}$ can be rewritten as follows:

17 $$\begin{aligned} F_{penalty}= \frac{sign(x)-1}{2}\max \biggl ( \frac{T_{low}-I(x)}{T_{low}}, \frac{I(x)-T_{high}}{I(x)}\biggr ) \end{aligned}$$

$F_{penalty}$ is always negative for areas outside the valid region, indicating that pixels outside the region are assigned a penalty value. This term is, therefore, incorporated into the fitting term to guide the level set evolution toward deflation.

Minimizing the energy function in Eq. (7) with respect to $\phi$ by calculating the first variation, we obtain the evolution equation as follows:

18 $$\begin{aligned} \begin{aligned} \frac{\partial \phi }{\partial t}&= \lambda \delta \bigl ( \phi (x)\bigr ) \text {div} \biggl (\frac{\nabla \phi (x)}{|\nabla \phi (x)|}\biggr ) + \delta \bigl ( \phi (x) \bigr ) \int _{\Omega _y} K_\sigma (x,y) \nabla _{\phi (y)} F_{new}\bigl (I(y), \phi (y) \bigr ) \,dy\\ \end{aligned} \end{aligned}$$

In the level set method, $\phi$ is moved toward the boundary of a target object, like cartilage, on the condition that Eq. (18) is minimized in each step.

## Results

### Parameters and performance metric

We demonstrated the performance of the proposed method on cartilage segmentation in knee MR images with a size of $384 \times 384 \times 160$ pixels. We set $\lambda =0.008 \times 255 \times 255$ , $\alpha =8$ , $\beta =1$, Gaussian kernel size $\sigma =2$, $\Delta t=0.1$. Then, $T_{low}$ and $T_{high}$ were constructed from 20 cartilage samples to obtain $T_{low}=148$ and $T_{high}=233$. The proposed method was compared with the adjusted Lanktons method [13] that incorporated SFCM initialization in the same way as the proposed method. We used Advanced Normalization Tools (ANTs) [18] to create template images of prior information from 20 participants in the progression sub-cohort from the OAI. To validate the performance comparisons, we used DSC to show the similarity between a test image and the reference image. A higher DSC value indicates better agreement between two binary masks:

19 $$\begin{aligned} DSC=\frac{2|A \cap M|}{|A| + |M|} \end{aligned}$$

where *A* represents the automatic segmentation mask and *M* is the manual segmentation by experts, which is the standard for comparison. In this paper, we use average DSC for segmentation accuracy, because one subject has 256 slices.

## Level set based algorithm

**Fig. 3:**
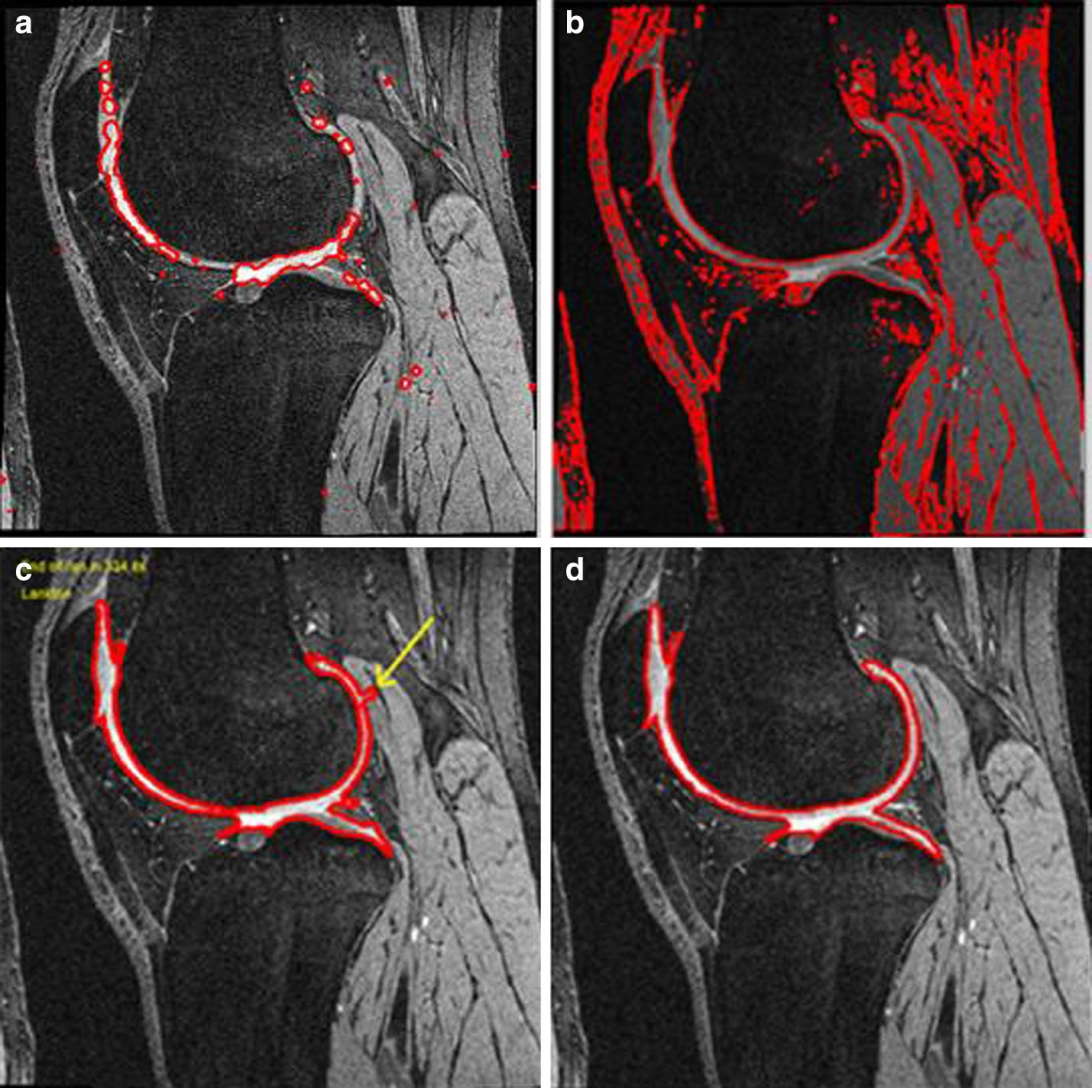
Cartilage segmentation in knee MRI. **a** Edge-based level set based cartilage segmentation in knee. **b** Global region region-based level set. **c** Localizing region-based level set. **d** Referenced manual segmentation

We performed a preliminary experiment of cartilage segmentation from a knee MRI with the existing level set segmentation, edge-based level set segmentation, and segmentation to localize active contours. Figure 3 shows the results: Fig. 3a is the edge-based level set [12], Fig. 3b is the global region-based level set, Fig. 3c is the localizing region-based level set [13], and Fig. 3d is the manual segmentation, used as the standard for evaluating automatic segmentation. Figure 3a shows the results of automatic edge-based level set segmentation, which automatically sets the initial contour with a spatial fuzzy clustering technique.

The results show that the edge-based level set algorithm is not suitable for segmenting knee cartilage, because the knee cartilage is inhomogeneous and has a weak boundary. Figure 3c shows the results of localizing region-based level set segmentation, which had sufficient resolution to better extract inhomogeneous objects when compared with the global region-based level set segmentation in Fig. 3b. Localizing region-based level set segmentation, however, depends on the position of the initial contour. If the initial contour is close to the knee cartilage, the result is more accurate, but if not, the result is poor. Since the initial contour included some muscle pixels, the result includes some muscle tissue, like the region marked in yellow in Fig. 3c. The results of Fig. 3 indicate that an algorithm is required to calculate the initial contour, and a new force function is needed for more accuracy in the level set algorithms.

**Fig. 4:**
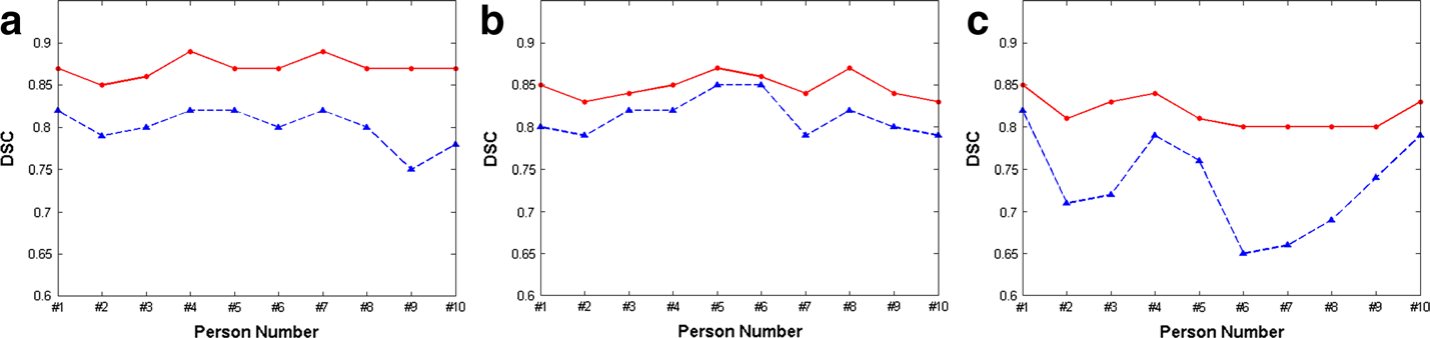
Overlap analysis of the proposed segmentation (*solid line*) and Lanktons segmentation (*dashed line*). **a** Average DSC of femoral cartilage. **b** Average DSC of tibial cartilage. **c** Average DSC of patellar cartilage

Table 2 shows the performance of the proposed segmentation via comparison with the gold standard that is provided by experts. The average DSCs for the femoral, patellar, and tibial cartilage from 10 people, that are different from 20 subjects for template data, were 0.871, 0.817 and 0.848, with standard deviations of 1.10, 1.40 and 1.79 %, respectively. We compare the average DSCs of the proposed segmentation with Lankton’s segmentation in Fig. 4. The proposed method showed improvements of 8.8, 4.3 and 3.5 % in the average DSCs for the femoral, tibial and patellar cartilage, respectively.

**Table 2:** DSC results with standard deviations from 10 people with 160 slices each

Person no.	Femoral cartilage DSC	Patellar cartilage DSC	Tibial cartilage DSC
#1	0.87 (1.80 %)	0.85 (2.86 %)	0.85 (2.29 %)
#2	0.85 (0.52 %)	0.81 (0.86 %)	0.83 (2.36 %)
#3	0.85 (1.00 %)	0.83 (2.47 %)	0.84 (2.87 %)
#4	0.89 (1.60 %)	0.84 (3.32 %)	0.85 (1.50 %)
#5	0.87 (1.88 %)	0.81 (0.83 %)	0.87 (3.77 %)
#6	0.87 (1.74 %)	0.80 (1.29 %)	0.86 (2.18 %)
#7	0.89 (0.91 %)	0.80 (1.11 %)	0.84 (1.51 %)
#8	0.87 (1.04 %)	0.80 (2.89 %)	0.87 (3.60 %)
#9	0.87 (1.29 %)	0.80 (0.99 %)	0.84 (1.16 %)
#10	0.87 (1.39 %)	0.83 (1.75 %)	0.83 (1.30 %)

**Fig. 5:**
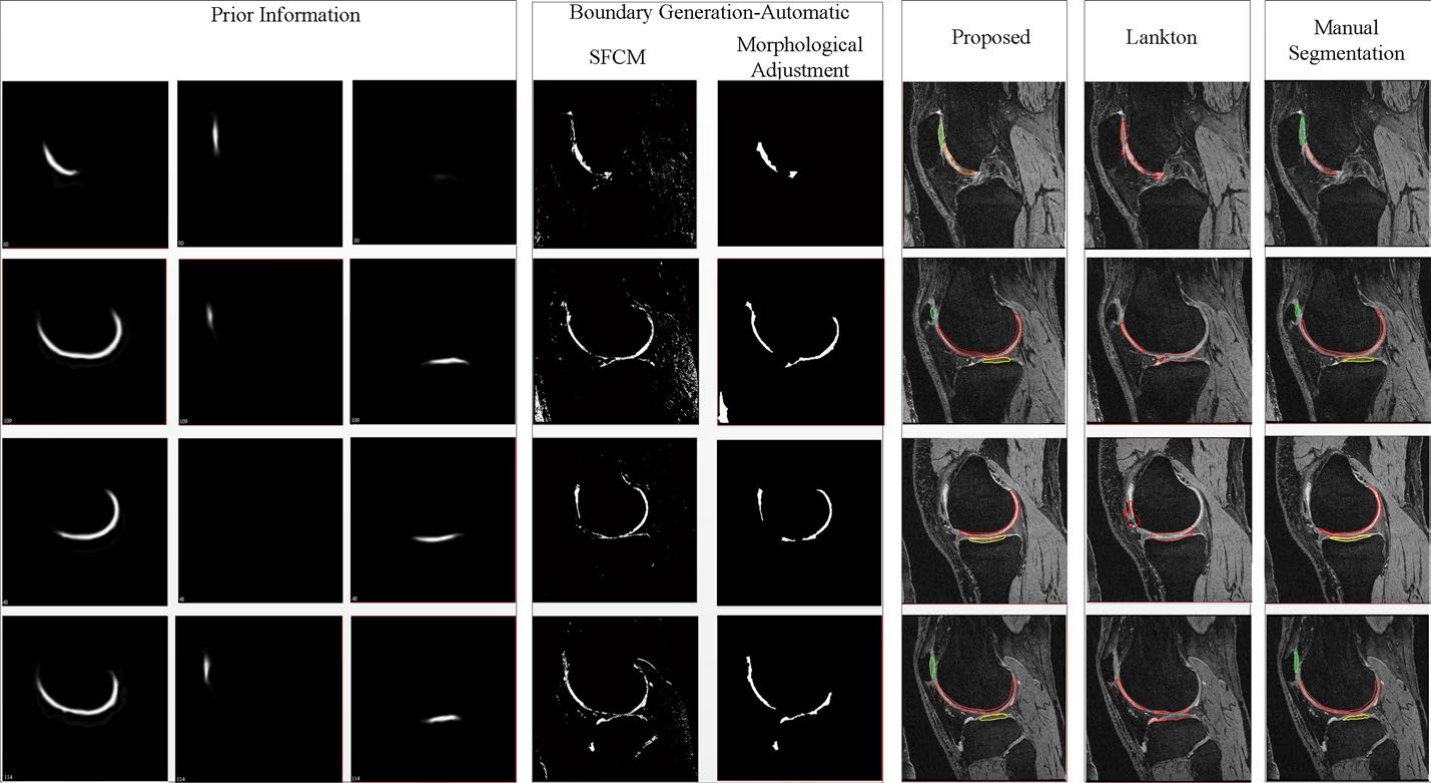
Knee cartilage segmentation for various cases in two dimensions. Each *row* represents a slice of a knee MR image in a single series. **a** Our template image from normal subjects: femoral (*left*), patellar (*center*), and tibial (*right*) cartilage. **b** Warping results from the template to the original image: SFCM (*left*) and morphological adjustment (*right*). **c** The result of Lanktons method. **d** The result of our proposed method. **e** The result of manual segmentation for reference

We collected three types of template data: femoral, patellar, and tibial cartilage templates. Our templates were based on manual segmentation from 20 normal people. Figure 5a shows template data images of femoral, patellar, and tibial cartilage. In the first row, only the femoral and tibial cartilage can be distinguished; in the second row, only the femoral and patellar cartilage are distinguishable; the third and fourth rows represent femoral, patellar, and tibial cartilage. Our method uses the integrating SFCM to approximate the cartilage contours in Fig. 5b. We then adjust morphological operators to remove unnecessary small areas from the SFCM result. Figure 5b shows that the morphological adjustment efficiently identified the ROI for the initial contour of the level set algorithm. Lanktons method uses a manual initial boundary, which is almost identical to the manual segmentation boundary. The results of Lankton’s method in Fig. 5c indicate that the femoral, patellar, and tibial cartilage cannot be differentiated, because Lanktons method does not use template data. Our proposed method uses template data not only in the initial boundary decision step, but also in the level set force function. The proposed method uses an energy function of knee template data, $F_{template}$, in Eq. (9), and distinguishes each of the femoral, patellar, and tibial cartilage boundaries.

For example, if $F_{template}$ in Eq. (9) is the template data of the femoral cartilage, then the result of our segmentation is the red boundary in Fig. 5d. If we apply the template data for the patellar or tibial cartilage to the $F_{template}$ in Eq. (9), the result is the green or yellow boundary, respectively, in Fig. 5d. Figure 5e shows the manual segmentation used as the basis for comparison. The perceptual evaluation in Fig. 5, which combines segmentation results of different structures denoted by different color assignments for visual assessment, demonstrates that our proposed method is more precise than Lankton’s method, our reference method. Lankton’s method could not segment each cartilage as a separate object.

## Recent knee segmentation method

The recent method, introduced by Tamez-Pena et al. [6], is based on the fuzzy voting algorithm and has the best segmentation performance to the best of our knowledge. We select it as the reference paper for performance comparison of our works. Table 3 shows a comparison of three segmentation methods: a recent knee segmentation method introduced by Tamez-Pena et al. [6], a reference segmentation by Lankton [13], and our proposed method. All three segmentation methods had a high specificity of methodology (>99 %) and at least 87 % sensitivity. Overlap analysis of our proposed segmentations showed an increase in accuracy of 7, 3 and 8 % as compared with Lankton’s method at the femur, tibia, and patella, respectively. We also compared the proposed method with Tamez-Pena et al.’s method, which is based on a fuzzy voting algorithm using six normal subjects. Tamez-Pena et al.’s approach includes a proprietary atlas which was unavailable to public. Thus, we used our own template data and implemented the Tamez’s algorithm. The average overlap accuracy was 89 % at the tibial cartilage and 88 % at the femoral cartilage, and there was no data for patellar cartilage. Our segmentation is based on a widely used level-set algorithm. We used 20 normal subjects from the OAI datasets for our template data, as did Tamez-Pena et al. However, our template data included prior information from 20 normal subjects, whereas Tamez-Pena et al.’s method does not use prior information. We obtained DSC values of 87.1, 84.8 and 81.7 % for the femoral, tibial, and patellar cartilage, respectively, with lower standard deviations than those seen in Tamez-Pena et al.’s method. Also, the sensitivities of our results were 90.6 % for femoral cartilage and 87.5 % for tibial cartilage, which were higher than those seen in Tamez-Pena et al.’s method (88 and 89 %, respectively).

**Table 3:** DSC comparisons with standard deviations

	Segmented tissue	DSC	Sensitivity	Specificity
A recent knee segmentation [6]	Femoral cartilage	88.0 % (4.0 %)	88.0 % (4.00 %)	99.9 % (0.00 %)
	Tibial cartilage	84.0 % (5.0 %)	89.0 % (6.00 %)	100 % (0.00 %)
	Patellar cartilage	–	–	–
The localizing region-based active contour segmentation [13]	Femoral cartilage	80.0 % (2.14 %)	99.9 % (0.00 %)	99.9 % (0.00 %)
	Tibial cartilage	81.3 % (2.19 %)	99.9 % (0.00 %)	99.9 % (0.00 %)
	Patellar cartilage	73.3 % (5.44 %)	99.9 % (0.00 %)	99.9 % (0.00 %)
The proposed segmentation	Femoral cartilage	87.1 % (1.10 %)	90.6 % (2.22 %)	99.7 % (0.05 %)
	Tibial cartilage	84.8 % (1.40 %)	87.5 % (3.82 %)	99.9 % (0.01 %)
	Patellar cartilage	81.7 % (1.79 %)	90.2 % (1.24 %)	99.8% (0.04 %)

## Discussion

The purpose of our study is to improve the segmentation performance for knee cartilages and to approach with level set based segmentation method which is effective in field inhomogeneity, i.e., pixel intensities have a great deal of variation within the same tissue. Femur bone, tibia bone, and cartilage all suffer difficulty in segmentation from field inhomogeneity.

Above all, we make template images for each cartilage such as femoral, tibial, and patellar cartilage, which is made by 5120 slices. To form the template data, we use SyGN for registration. The template images are applied to the automatic initial contour and the new energy function. In our previous work, we suggested SFCM algorithm to be a pre-processing step to approximate cartilage contours. SFCM can make initial contour for our proposal cartilage segmentation method. In this work, the proposed method is the first approach by level set method for knee cartilage segmentation. We attempt to apply the localizing region-based active contours algorithm [13] into knee MRIs. However, it does not work because femoral, tibial, and patella cartilage are too thin to directly adjust the localizing active contours algorithm. To overcome this problem, we make template images for each cartilage and modify force function in level set algorithm. As a result, our proposed algorithm overcomes the problem of thin and inhomogeneity with improving segmentation performance. We evaluate the performance of segmentation accuracy by DSC. We compare with referenced Lanktons method [13]. Actually, both methods are synchronized automatic initial contour and the template data. The only difference is the force function in level set algorithm. But we improved 8.8, 4.3 and 3.5 % in the average DSCs for femoral, tibial, and patellar cartilage, respectively. And we perform the evaluation of the recent knee segmentation method and our proposal method. Actually, we evaluate the similar accuracy of the cartilage segmentation with lower standard deviations than Tamez-Pena et al.’s method. Also, the sensitivity of our results were 90.6 and 87.5 % for femoral cartilage and tibial cartilage, compared with Tamez-Pena et al.’s method (88 and 89 %, respectively).

Regarding execution time, we spend about 180 h to make the template data by using group-wise registration, SyGN [17], with the exception of the manual segmentation time. It takes additional 48 h to segment all cartilages from one’s MRI data set in our experimental environment. Our experiment was conducted in dual CPUs (Intel Xeon 3.0GHz × 2) and 16GB dram hardware with Red Hat 4.4.5 linux.

## Conclusions

We propose a fully-automated, level set-based knee segmentation with a template (or atlas prior). For a fully automation, we apply SFCM to create an initial contour automatically. The manually segmented data by experts are used to construct the template data. The template data had a significant effect on the result of cartilage segmentation, which led to improved performance of our approach. We modify Lankton’s force function based on the level set algorithm [13] to improve its accuracy for knee cartilage segmentation. The modified force function supplements the energy function of the template and penalty in Lankton’s force function. Moreover, we create a template dataset with MRI data from 20 normal subjects from the OAI database. The SFCM algorithm used the template for full automation, and the modified force function also used the template to improve segmentation accuracy. By using multiple thresholds as global information, the proposed method overcomes the limitations of Lankton’s method. The experimental results show that the proposed method increases performance by an average of 5 % over Lankton’s method. Our proposed method provides a fully automatic and robust model for knee cartilage segmentation.

Our method has some limitations; the modified force function is based on slice-by-slice processing and the performance is significantly affected by the template dataset. For further studies, we will use a full 3D segmentation method to improve performance.

## Abbreviations

OA: osteo arthritis
OAI: osteo arthritis initiative
FCM: Fuzzy C-Mean
SFCM: Spatial Fuzzy C-Mean
DSC: dice similarity coefficient
MRI: magnetic resonance imaging
MR: magnetic resonance
CT: computed tomography
3-D: three-dimensions
3-T: 3-Tesla
DESS: dual echo steady state
WE: water excitation
SyGN: Symmetric Group-wise Normalization
ROI: region of interest
C–V: Chan-Vese
ANTs: advanced normalization tools
CPU: central processing unit
